# Loss of circRNAs from the *crh‐1* gene extends the mean lifespan in *Caenorhabditis elegans*

**DOI:** 10.1111/acel.13560

**Published:** 2022-01-31

**Authors:** David Knupp, Brian G. Jorgensen, Hussam Z. Alshareef, Jaffar M. Bhat, Jeremy J. Grubbs, Pedro Miura, Alexander M. van der Linden

**Affiliations:** ^1^ Department of Biology University of Nevada, Reno Reno Nevada USA; ^2^ Present address: Department of Botany Government Degree College Kupwara Jammu & Kashmir India

**Keywords:** aging, *Caenorhabditis elegans*, circRNA, *crh‐1*, reverse complementary match

## Abstract

Accumulation of circular RNAs (circRNAs) during aging occurs on a genome‐wide level for multiple organisms, but its significance is unknown. Generating circRNA loss‐of‐function mutants is difficult because the vast majority of these RNAs are comprised of exons shared with protein‐coding mRNAs. In *Caenorhabditis elegans*, most circRNAs were previously found to accumulate during aging. Two of the most abundant, age‐accumulating circRNAs are generated from exon 4 of the *crh*‐*1* gene (circ‐*crh*‐*1*). Here, we found that the biogenesis of circ‐*crh*‐*1* was regulated by the double‐stranded RNA‐binding protein ADR‐1. We identified Reverse Complementary Match (RCM) sequences in introns flanking circ‐*crh*‐*1*. Using CRISPR‐Cas9, we deleted the downstream RCM and found that this completely eliminated expression of the circRNA without affecting linear mRNA expression from the *crh*‐*1* gene. Remarkably, worms lacking circ‐*crh*‐*1* exhibited a significantly longer mean lifespan. Lifespan was partially restored to wild type by expression of circ‐*crh*‐*1* in neural tissues. Widespread transcriptome alterations in circ‐*crh*‐*1* mutants were identified using RNA‐Seq. Moving forward, intronic RCM deletion using CRISPR should be a widely applicable method to identify lifespan‐regulating circRNAs in *C*.* elegans*.

## Graphical abstract

Disruption of intronic RNA–RNA base‐pairing (RCM) by CRISPR‐Cas9 abolishes circRNA expression from the *crh‐1*/CREB gene in *Caenorhabditis elegans* without affecting host‐gene mRNA or protein expression. circ‐*crh‐1* deficient worms exhibit significant extension in mean lifespan. This is the first lifespan phenotype attributed to the loss of a circRNA.

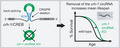

**Abbreviations**
circRNA: circular RNA
CREB: cAMP response element‐binding protein
CRISPR: Clustered Regularly Interspaced Short Palindromic Repeats
DEG: differentially expressed genes
mRNA: messenger RNA
RCM: reverse complementary Match
RNA‐Seq: RNA sequencing

## 1 INTRODUCTION, RESULTS, AND DISCUSSION

**FIGURE 1:**
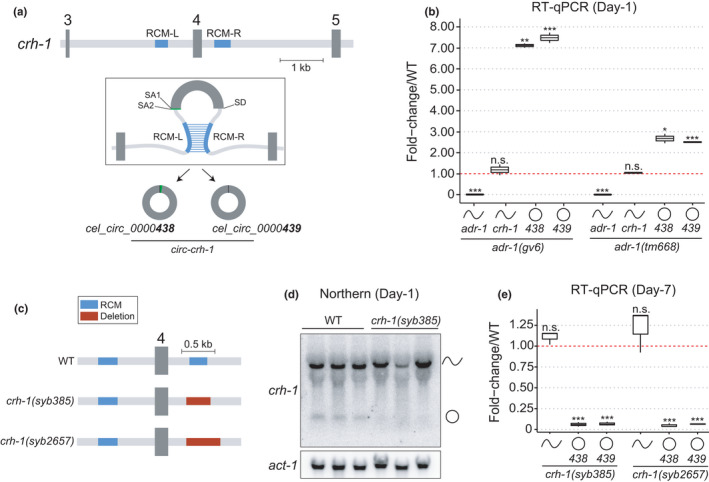
circ‐*crh*‐*1* regulation by ADR‐1 and generation of CRISPR/Cas9 deletion alleles. (a) Schematic showing exon 3 to exon 5 of *crh*‐*1* gene (chrIII:11685086–11691812). Two circRNAs are generated by backsplicing of exon 4, using two alternative splice acceptors (SA) and one shared splice donor (SD). Reverse complementary matches (RCM‐L and RCM‐R) predicted to facilitate backsplicing of *crh*‐*1* circRNAs are shown as blue rectangles. (b) Mutations in *adr*‐*1* result in increased expression of circ‐*crh*‐*1* but not linear *crh*‐*1* in day‐1 adult worms, as determined by RT‐qPCR. Linear *adr*‐*1* transcripts are not expressed in *adr*‐*1* *(* *gv6* *)* or *adr*‐*1* *(* *tm668* *)* mutant alleles as expected. *n* = 2 independent biological samples. (c) Schematic of *crh*‐*1* exon 4 and flanking intronic sequences from either wild‐type (WT) or *crh*‐*1* circRNA mutant genotypes. Intronic deletions targeting the downstream RCM‐R region were introduced by CRISPR/Cas9 and are presented as red rectangles. (d) Northern analysis of day‐1 adult whole worms using dsDNA probe complementary to *crh*‐*1* exon 4. Signal from *crh*‐*1* circRNAs is absent in *crh*‐*1(syb385)* mutant worms compared to wild type. (e) RT‐qPCR expression analysis of linear and circular *crh*‐*1* transcripts in day‐7 adult *crh*‐*1* circRNA mutants compared to wild‐type worms. Both circRNAs are significantly reduced compared to wild type, whereas the *crh*‐*1* linear RNA is unchanged. *n* = 3 independent biological samples. For RT‐qPCR expression analyses, data were normalized to *cdc42* mRNA and are represented as mean ± SEM; n.s., not significant; *p < 0.05; **p < 0.01; ***p < 0.001. Strains used in this study are found in Table S1. RT‐qPCR and northern blot primers can be found in Table S2. Also see Figure S1

CircRNAs are a recently appreciated class of RNAs generated by backsplicing (Li et al., 2018). Most characterized circRNAs are produced from exons of protein‐coding genes (Zhang et al., 2014). CircRNAs lack free ends, resulting in greater resistance to exoribonuclease digestion compared to their linear RNA counterparts (Jeck et al., 2013). Interestingly, circRNAs were found to accumulate in the brains of *Drosophila* and rodents during aging (Gruner et al., 2016; Jeck et al., 2013; Zhou et al., 2018). Previously, we demonstrated that the majority of circRNAs expressed in *Caenorhabditis elegans* also accumulate during aging and that several age‐accumulated circRNAs are generated from genes with known roles in lifespan regulation (Cortes‐Lopez et al., 2018). One of these genes, *crh*‐*1*, encodes an ortholog of the cAMP Response Element‐Binding Protein (CREB) which plays a role in longevity (Chen et al., 2016; Lakhina et al., 2015). We previously reported that the two abundant circRNAs generated from the *crh*‐*1* gene greatly increase in abundance during aging (i.e., *cel_circ_0000438* and *cel_circ_0000439*) (Cortes‐Lopez et al., 2018). These circRNAs differ only in six nucleotides as a result of an alternative splice acceptor site and are collectively referred to from here on as circ‐*crh*‐*1* (Figure 1a).

For certain circRNAs, RCMs within flanking introns facilitate backsplicing ostensibly by bringing the splice donor and acceptor sites into closer proximity (Jeck et al., 2013). The two *crh*‐*1* circRNAs generated from exon 4 of the *crh*‐*1* gene are flanked by long introns that contain sequences complementary to one another (RCM‐L and RCM‐R) (Figure 1a, Figure S1a). ADAR is a double‐stranded RNA‐binding protein that when knocked down increases the expression of some circRNAs in mammalian cells (Ivanov et al., 2015; Rybak‐Wolf et al., 2015). We investigated *crh*‐*1* expression in two independent ADAR null mutant alleles, *adr*‐*1(gv6)* and *adr*‐*1(tm668)* (Hundley et al., 2008; Tonkin et al., 2002). RT‐qPCR analysis of whole worms showed that circ‐*crh*‐*1* expression was significantly increased in both *adr*‐*1* mutants, whereas linear *crh*‐*1* mRNA was unchanged (Figure 1b). These data demonstrate that ADR‐1 negatively regulates circ‐*crh*‐*1*, most likely through interacting with intronic RCMs.

Generating circRNA loss‐of‐function organisms is challenging because circRNAs are derived from protein‐coding genes, and thus attempts to disrupt circRNA expression can interfere with the biogenesis of protein‐coding transcripts. Recently, CRISPR/Cas9 was used to delete RCMs in the introns flanking mouse circ*Kcnt2* which abolished the circRNA and did not affect the linear RNA (Liu et al., 2020). We similarly used CRISPR/Cas9 to generate a 377 bp deletion overlapping the RCM‐R region to generate the *crh*‐*1(syb385)* mutant strain (Figure 1c). To confirm the loss of circ‐*crh*‐*1*, we performed northern blot analysis and found circ‐*crh*‐*1* to be undetectable, whereas linear *crh*‐*1* expression persisted (Figure 1d). By RT‐qPCR analysis, circ‐*crh*‐*1* expression was barely detectable in *syb385* mutants in both 1‐ and 7‐day‐old adult worms (Figure 1e and Figure S1b). Importantly, we found that expression of linear *crh*‐*1* was not significantly affected (Figure 1e and Figure S1b). In addition, we generated *adr*‐*1(gv6)*; *crh*‐*1(syb385)* double mutants and examined whether suppression of circ‐*crh*‐*1* by ADR‐1 depends on RCM base‐pairing. We found that in contrast to *adr*‐*1(gv6)* mutants, circ‐*crh*‐*1* expression in these double mutants remained unchanged compared to *crh*‐*1(syb385)* (Figure 1b and Figure S1b). Together, this suggests that RCMs are likely required for ADR‐1 regulation of circ‐*crh*‐*1* biogenesis. Next, we generated a second independent allele, *crh*‐*1(syb2657)*, which was designed to delete a slightly larger portion of the intronic region surrounding the RCM‐R sequence (Figure 1c), and confirmed by RT‐qPCR that only the circle and not mRNA from the *crh*‐*1* gene was altered (Figure 1e and Figure S1c). These two alleles, thus, represent genuine circRNA‐specific loss‐of‐function mutants.

**FIGURE 2:**
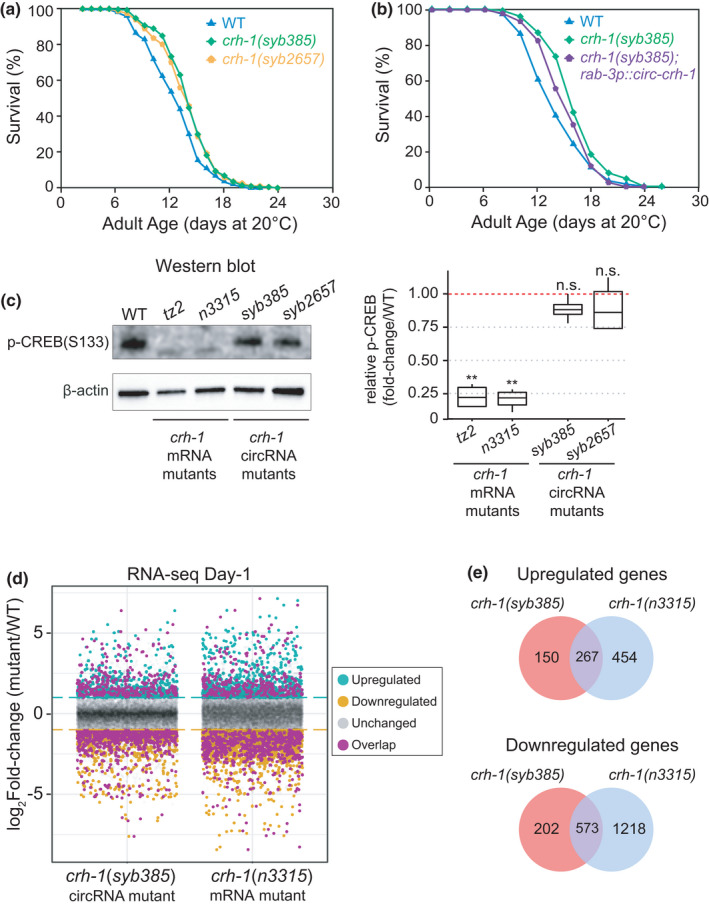
Loss of circ‐*crh1* extends *Caenorhabditis elegans* lifespan and alters the transcriptome. (a) Loss of *crh*‐*1* circRNAs extends the mean lifespan. Lifespan curves for *crh*‐*1(syb385)* mutants (11.5% increase vs. wild type, *p* < 0.0001, Mantel‐Cox log‐rank test) and *crh*‐*1(syb2657)* mutants compared to wild type (9.8% increase vs. wild type, *p* < 0.001, Mantel‐Cox log‐rank test). *n* = 3 independent lifespan assays were performed with *n* > 150 animals for each assay and genotype in the absence of FUdR (see Supporting Information). (b) Lifespan curves for worms overexpressing circ‐*crh*‐*1* in *rab*‐*3*‐expressing neurons compared to wild‐type and *crh*‐*1(syb385)* mutants. There is a non‐significant difference in mean lifespan between wild‐type and *crh*‐*1(syb385)*; *rab*‐*3p*::*circ*‐*crh*‐*1* transgenic worms (*p* = 0.0131; Mantel‐Cox log‐rank test), as well as between *crh*‐*1(syb385)* and *crh*‐*1(syb385)*; *rab*‐*3p*::*circ*‐*crh*‐*1* transgenic worms (*p* = 0.0456; Mantel‐Cox log‐rank test). *n* = 2 independent lifespan assays were performed with *n* > 100 animals for each assay and genotype in the absence of FUdR (see Supporting Information). See Table S3 for lifespan statistics. (c) (*Left*) Phosphorylated CRH‐1 protein levels were absent in *crh*‐*1* null or loss‐of‐function mutants (*tz2*, *n3315*) but unaffected in *crh*‐*1* circRNA mutants (*syb385*, *syb2657*). A representative western blot is shown. The signal indicates p‐CRH‐1 using an antibody directed against mammalian p‐CREB (Ser 133) (top panel). ß‐actin loading control is shown below. (*Right*) Quantification of p‐CREB expression in 1‐day‐old adult worms. Data are normalized to wild type and represented as the mean ± SEM; n.s., not significant, **p < 0.01; *n* = 4 independent biological replicates. (d) RNA‐Seq analysis showing mRNA expression changes in *crh*‐*1(syb385)* and *crh*‐*1(n3315)* versus wild‐type day 1 adult worms. Significantly downregulated and upregulated genes (Log_2_ fold‐change >2, adj. *p* < 0.05) are shown as orange and green dots, respectively. Genes that were differentially expressed in both genotypes relative to wild‐type worms are shown as purple dots. *n* = 4 biological replicates per genotype. (e) Overlap of differentially expressed genes between *crh*‐*1(syb385)* and *crh*‐*1(n3315)* mutants versus wild‐type worms. Also see Figure S2

To directly test whether circ‐*crh*‐*1* plays a role in aging, we performed lifespan experiments at 20ºC for both *crh*‐*1(syb385)* and *crh*‐*1(syb2657)* mutant worms. Hundreds of circRNAs dramatically increase in expression during aging (Cortes‐Lopez et al., 2018). Remarkably, the removal of just circ‐*crh*‐*1* caused a significant extension of mean lifespan compared to wild‐type controls for both alleles (*syb385*, 11.5% extension, *p* < 0.0001; *syb2657*, 9.8% extension, *p* < 0.001) (Figure 2a, Table S3). This suggests that circ‐*crh*‐*1* might contribute to age‐related decline. Expression of circ‐*crh*‐*1* under control of the germline promoter *pie*‐*1* or the pan‐neural promoter *rab*‐*3* restored circ‐*crh*‐*1* expression levels in *syb385* mutants (Figure S2a,b,d). Expression of circ‐*crh*‐*1* driven by *rab*‐*3*, but not *pie*‐*1*, was able to partially rescue the lifespan phenotype observed in *syb385* mutants (Figure 2b, Figure S2c and Table S3). These results indicate that circ‐*crh*‐*1* expression in neurons is an important determinant of *C*. *elegans* lifespan.

To demonstrate that lifespan extension observed in *crh*‐*1* circRNA mutants was independent of CRH‐1 protein expression, we measured phosphorylated CRH‐1 protein (p‐CREB) via western blot. As expected, p‐CREB was undetectable in null *crh*‐*1(n3315)* and *crh*‐*1(tz2)* mRNA mutants (Figure 2c). p‐CREB expression was not altered in circ‐*crh*‐*1* mutants compared to wild type (Figure 2c). Similarly, we observed that the expression of circ‐*crh*‐*1* was unchanged in the p‐CREB protein null condition (Figure S2e). Together, these data suggest that p‐CREB is not regulated by circ‐*crh*‐*1* and likewise, CREB does not regulate circ‐*crh*‐*1* biogenesis. However, we cannot eliminate the possibility that circ‐*crh*‐*1* and CREB might regulate one another at particular timepoints or in specific tissues not examined here.

As previously reported (Chen et al., 2016; Templeman et al., 2020), p‐CREB mRNA mutants (*n3315* and *tz2*) are short‐lived, whereas we observed a longer mean lifespan for circ‐*crh*‐*1* mutants (Figure 2a). In order to identify transcriptomic differences that might contribute to the opposing lifespan phenotypes in p‐CREB mRNA mutants versus circ‐*crh‐1* mutants, we turned to RNA‐Seq analysis. We sequenced 1‐day‐old adult worms from *crh*‐*1(syb385)* and *crh*‐*1(n3315)* mutant backgrounds (Table S4) and identified hundreds of differentially expressed genes (DEGs) when compared to wild‐type controls (Figure 2d and Table S5). To our surprise, we discovered a significant overlap (*p* < 0.0001; Fisher's exact test) for both upregulated and downregulated genes between *syb385* and *n3315* mutant alleles (Figure 2d,e). Gene ontology analysis of the 267 shared upregulated genes showed significant enrichment of collagen‐encoding genes (Figure 2e and Table S6). Upregulation of collagens is a shared feature found in several different long‐lived mutants (Ewald et al., 2015). While this finding is supportive of the increased lifespan observed in *crh*‐*1(syb385)* mutants described here, *crh*‐*1(n3315)* mutants have a reduced lifespan. Hence, other differentially expressed genes unique to each mutant genotype (e.g., non‐overlapping DEGs) are likely responsible for determining *C*. *elegans* overall lifespan.

CircRNAs generally increase in expression during aging, which has been attributed to their high degree of stability, especially in post‐mitotic tissues such as neurons (Knupp & Miura, 2018). We have speculated previously that the age accumulation of circRNAs could be detrimental to cellular function due to the progressive nature of the age accumulation (Knupp & Miura, 2018). Given the large number of circRNAs increased with aging, perhaps such a detrimental effect is independent of the specific identity of the individual circRNAs. Despite our hypothesis that circRNA accumulation could decrease lifespan, it nonetheless was quite surprising to find that loss of a single circRNA can increase the mean lifespan. Which of the other hundreds of age‐accumulated circRNAs might impact lifespan? Given the enrichment of intronic RCMs near *C*. *elegans* circRNA loci (Cortes‐Lopez et al., 2018), the relative ease of CRISPR gene editing, and the general utility of *C*. *elegans* in aging research, screening additional age‐associated circRNAs appears to be a logical next step.

Some age‐accumulated circRNAs might have beneficial roles in aging cells. Recently, circSfl transgenic overexpression was found to extend lifespan in *Drosophila* (Weigelt et al., 2020). However, siRNA knockdown of circSfl was unsuccessful, and genetic manipulations performed to reduce the circRNA also impacted linear mRNA isoforms. This highlights the difficulties in generating circRNA loss‐of‐function mutants to study their impact on aging. In vivo siRNA knockdown has been successfully implemented for certain circRNAs in *Drosophila* (Pamudurti et al., 2020), but some circRNAs simply cannot be specifically targeted due to sequence limitations of the back‐spliced junction region. *C*. *elegans* have generally shorter introns than *Drosophila* and mammalian model systems. This might make them more amenable to efficient CRISPR manipulation of RCMs; however, it remains to be seen whether many *C*. *elegans* circRNAs can be specifically reduced or abolished using the RCM deletion methodology.

## 2 METHODS

A full description of methods can be found in the Supporting Information. Briefly, *C*. *elegans* were cultivated on the surface of NGM agar seeded with the *Escherichia coli* strain OP50 and grown in 20°C incubators using standard protocols. CRISPR deletion mutants were generated by Suny Biotech. Lifespan analysis was carried out with synchronized adult N2 or outcrossed mutant worms with or without transgenes at 20°C. Lifespan curves were analyzed using OASIS2 (Han et al., 2016). Raw RNA‐Seq reads are deposited at GEO (GSE190124). RNA‐Seq alignment was performed using STAR v2.7.5a (Dobin et al., 2013) and differential expression was performed using DESeq2 (Love et al., 2014).

## CONFLICT OF INTEREST

The authors declare no competing interests.

## AUTHOR CONTRIBUTIONS

DK, BGJ, PM, and AVDL designed the study; DK, BGJ, HZA, JMB, and JJG performed the experiments. DK, BGJ, HZA, PM, and AVDL analyzed the data. DK, BGJ, PM, and AVDL wrote the manuscript. AVDL and PM supervised the study.

## Supporting information

Supplementary Material

## Data Availability

The data that support the findings of this study are available in the Supporting Information of this article. The RNA‐Seq data are deposited at the Gene Expression Omnibus (GEO) under GSE190124.
