# Supplementary material for: Loss of circRNAs from the crh‐1 gene extends the mean lifespan in Caenorhabditis elegans
Source: Aging Cell. 2022 Jan 31;21(2):e13560. doi: 10.1111/acel.13560 (PMC8844124; doi:10.1111/acel.13560)
Supplement: Supplementary file 1 — Supplementary Material [file ACEL-21-e13560-s001.docx]

**Elimination of circRNAs from the *crh-1* gene extends mean lifespan in *C. elegans***

**Supporting Information**

**Figure S1. Intronic RCMs flanking *crh-1* exon 4 mediate expression of circ-*crh-1* and regulation by ADR-1.** (a) Sequence alignment of RCM-L and RCM-R flanking exon 4 of the *crh-1* gene. (b) RT-qPCR expression analysis of linear and circular *crh-1* transcripts in day-1 *crh-1(syb385),* *adr-1(gv6)*, and *crh-1(syb385);adr-1(gv6)* adult worms compared to wild-type. Both circRNAs are significantly reduced in *crh-1(syb385)* and *crh-1(syb385);adr-1(gv6)* mutants whereas the linear *crh-1* transcript is unchanged. (c) RT-qPCR expression analyses were repeated for day-1 *crh-1(syb385)* and *crh-1(syb2657)* mutants relative to wild-type. In both genotypes, only the circRNA expression was significantly changed. For RT-qPCR, data was normalized to *cdc-42* and is represented as mean ± SEM; n.s., not significant; *, *P*<0.05; **, *P*<0.01; ***, *P*<0.001; n=3 independent biological replicates.

**Figure S2. Expression of circ-*crh-1* in molecular rescue or CREB null genetic backgrounds.** (a) Schematic of plasmid-based minigene used to overexpress circ-*crh-1* under the control of a user-defined promoter. (b) RT-qPCR expression analysis of linear and circ-*crh-1* transcript in the *crh-1(syb385); pie-1p::circ-crh-1* genetic backgrounds relative to wild-type worms at day-1. n=3 independent biological replicates (c) Lifespan curves for worms overexpressing circ-*crh-1* in *pie-1*-expressing germline cells compared to wild-type and *crh-1(syb385)* mutants. There was a non-significant difference in mean lifespan between *crh-1(syb385); pie-1p::circ-crh-1* and *crh-1(syb385)* mutants (*P*=0.8529, Mantel-Cox log-rank test) (Supplementary Table S3). n=3 independent lifespan assays were performed with n>100 animals for each assay and genotype in the absence of FUdR. (d) RT-qPCR expression analysis of linear and circ-*crh-1* transcript in the *crh-1(syb385); rab-3p::circ-crh-1* genetic backgrounds relative to wild-type worms at day-1. n=3 independent biological replicates. (e) circ-*crh-1* expression is not significantly different from wild-type in the CREB null background, *crh-1(tz2)* at day-1. n=3 independent biological replicates. For RT-qPCR expression analyses, data in panel (b,d) was normalized to *cdc-42*, while expression data in panel (e) was normalized to *act-1* mRNA and is represented as mean ± SEM; n.s., not significant; *, *P*<0.05; **, *P*<0.01.

**Supplementary Tables**

Supplementary Table S1: Strains used in this study.

Supplementary Table S2: Oligonucleotide primer information.

Supplementary Table S3: Summary and statistics of lifespan experiments.

Supplementary Table S4: RNA-Seq read statistics.

Supplementary Table S5: RNA-Seq expression analysis

Supplementary Table S6: DAVID GO enrichment analysis

**Supplemental Methods**

**Maintenance and handling of *C. elegans* strains**

Worms were cultivated on the surface of NGM agar seeded with the *E. coli* strain OP50, and grown in 20°C incubators using standard protocols. The wild-type strain N2, variety Bristol (Brenner, 1974) and other strains used in this study are listed in Supplementary Table S1. Strains generated and genotyped with respect to *crh-1* circRNA mutant alleles are listed in Supplementary Table S1.

**Generation of *crh-1* circRNA mutant alleles and transgenic animals**

Alleles with deletions in the predicted reverse-complementary match (RCM) intronic sequences flanking exon 4 of the *crh-1* locus (Figure 1c) were generated using a Co-CRISPR method (SunyBiotech). Generated *syb* mutant alleles were outcrossed to wild-type (N2) (Supplementary Table S1) and confirmed by PCR and Sanger sequencing. sgRNAs used to generate the *syb385* mutant were Sg1-ttaatctactctctgtaatctgg, Sg2-ccgcattgaaaatttccggcgaa, Sg3-ccgatttggcgattttttggtaa, Sg4-attctggcacattgccgatttgg, and for the *syb2657* mutant were Sg1-ttaatctactctctgtaatctgg, Sg2-ccggcaaactgcaatttggcaat, Sg3-atatccggcaaactgcaatttgg. To generate transgenic worms expressing the circ-*crh-1* in different tissues, exon 4 of *crh-1* and intronic sequences flanking exon 4 (see Figure S2a) were cloned into the pMC10 plasmid (a kind gift from the Sengupta Lab). Next, promoter sequences of *pie-1* (~1.1 kb) and *rab-3* (~1.2 kb) were cloned at the 5’-end of the circ-*crh-1* sequence using the multi cloning site (MCS) of pMC10. The generated constructs (i.e. *pie-1p::circ-crh-1*, *rab-3p::circ-crh-1*) were injected into wild-type (N2) at a concentration of 25-30 ng/μl along with *unc-122p*::RFP (AddGene) at a concentration of 75 ng/μl as a transgenesis marker to bring the final concentration up to ~100 ng/μl. Transgenic worms carrying extrachromosomal arrays overexpressing *pie-1p::circ-crh-1* or *rab-3p::circ-crh-1* were confirmed by RT-qPCR, and crossed with the six times outcrossed *crh-1(syb385)* mutant. Generated transgenic lines are listed in Supplementary Table S1.

**Reverse-complementary match (RCM) sequence analysis**

A RCM predicted to promote biogenesis of *crh-1* circRNAs (*cel_circ_0000439*, *cel_circ_0000438*) was identified from a pair of intron sequences flanking exon 4 of the *crh-1* gene using a custom script (<https://github.com/alexandruioanvoda/IntronPicker>) as described previously (Cortes-Lopez et al., 2018).

**Total RNA collection and extraction**

Worms were age-synchronized by hypochlorite treatment and collected eggs were hatched overnight at 20°C in 1x M9 buffer. L1 larvae were then plated onto NGM plates seeded with 10x concentrated *E. coli* OP50 bacteria and allowed to develop to the L4 larval stage at 20°C. L4 larvae were then collected, washed, and re-plated onto 25 µM 5-fluorodeoxyuridine (FUdR) (Milipore Sigma, Cat #50-91-9) containing NGM plates unless indicated otherwise seeded with 10x *E. coli* OP50 bacteria in order to prevent progeny formation. Adult worms were collected at different age-time points by filtering through a 30 µM nylon mesh to remove bacteria (Sefar, Cat #7050-1220-000-10). For RT-qPCR and RNA-seq analysis, aged adult worms were collected in Lysis/Binding Buffer from the mirVana miRNA isolation kit (Ambion, Cat #AM1560). Worms were immediately lysed by bead beating them for 5 min using a Bullet Blender Pro Storm (Next Advance). Total RNA was then extracted following mirVana manufacturers protocol and treated with DNAse I (Invitrogen, Cat #AM1907). For Northern analysis, 1-day old adult worms were collected using TRizol LS (ThermoFisher Scientific, Cat #10296028) and immediately frozen with liquid nitrogen. Lysates were freeze/thawed at −80°C, and disrupted with a Mixer Mill 400 (Retsch) and Dounce homogenizer (Corning) to break apart the cuticle of worms. Any cellular debris was removed by low-speed centrifugation. RNA was extracted using the Purelink RNA mini-kit with DNAse I treatment following the manufacturers protocol (Ambion, Cat #12183020). RNA samples for RNA-seq and Northern analysis were assessed by a Bioanalyzer (Agilent), and quantified using Quant-iT RiboGreen RNA Assay kit (ThermoFisher Scientific, Cat #R11490) and then stored at -80°C.

**RT-qPCR analysis**

To quantify and confirm individual circular or linear transcripts, 0.4 μg total RNA was reverse transcribed using Superscript III to prepare cDNA using random hexamers (Invitrogen, Cat #18080051). Next, cDNA samples were diluted and used with PowerUp SYBR Green Master Mix (Applied Biosystems, Cat #A25471) for RT-qPCR analysis analyzed on a CFX96 Real-Time System (Bio-Rad). For RT-qPCRs of circRNAs, we used outward facing primers. For host gene linear RNA counterparts, one primer was located in the circularizing exon and the other was located in the upstream or downstream non-circularizing exon. Fold-change values were calculated using wild-type (N2) ΔCt as control values for the 2^-ΔΔCt^ method. Data is normalized to *cdc-42* or *act-1* mRNA. Primer sequences are listed in Supplementary Table S2.

**Northern blot analysis**

For Northern analysis of circRNAs, 10 μg total RNA was used per sample and subsequently denatured using a deionized-glyoxal mixture for 1 hour at 55°C, then samples were put on ice for 5 min. Denatured samples were loaded onto a 1% BPTE+SeaKem LE Agarose (Lonza) gel and ran at 80V for 130 min. RNA samples were transferred to a Cytiva Whatman™ Nytran™ SuperCharge membrane (ThermoFisher Scientific, Cat #09-301-164) overnight using a Whatman™ TurboBlotter transfer system (ThermoFisher Scientific). Samples were then UV cross-linked with a Stratagene linker to the nylon membrane prior to probe hybridization. Double-stranded DNA probes were prepared by end-point PCR and labeled with dCTP [α-32P] (PerkinElmer, Cat # BLU513H250UC) using the Cytvia Amersham™ Megaprimer labeling kit (ThermoFisher Scientific, Cat #45-000-809) according to manufacture instructions. Blots were hybridized overnight in ULTRAhyb™ Ultrasensitive Hybridization buffer (ThermoFisher Scientific, Cat #AM8670) at 50°C. Following hybridization, blots were washed 2x10 min in a low-stringency buffer followed by 2x20 min in a high-stringency buffer at 50°C. Blots were then exposed for 4-5 days to a GE Storage Phosphor screen (Millipore Sigma) before imaging on a Typhoon™ FLA 7000 imager (GE). Probe sequences are listed in Supplementary Table S2.

**Mapping and quantification of RNA-seq datasets**

For RNA-seq analysis of linear transcripts, 1 μg total RNA was used from four independent biological replicates of wild-type (N2), *crh-1(syb385)* (VDL786) and *crh-1(n3315)* (MT9973) genotypes (Supplementary Table S4). Poly(A)-selected mRNA-seq libraries were prepared and sequenced on an Illumina HiSeq 6000 system to obtain paired-end 150nt reads at the University of California, Davis, genomics center by Novogene Co., LTD (Beijing, China). Reads were aligned to the WBcel235 reference genome using STAR v2.7.5a. FeatureCounts v1.5.0 (parameters -t exon -g gene_id) was used to obtain a counts table as input for differential expression analysis by DESeq2 (v1.26.0). A 2.0 fold-change and adj. *P*<0.05 was used to consider a linear RNA as differentially expressed (Supplemental Table S5). Raw read files are deposited in the GEOarchive repository (GSE190124). Individual accession numbers are listed in Supplementary Table S4.

**Gene ontology analysis**

To examine if certain sets of GO processes or keywords were enriched within the *crh-1(syb385)* or *crh-1(n3315)* vs wild-type differentially expressed gene set, we used the DAVID functional annotation clustering tool (Huang da et al., 2009). The Agilent *C. elegans* dataset was used as background. The results are shown in Supplementary Table S6.

**Western blot analysis of p-CREB levels**

To examine activated p-CREB levels, age-synchronized worms were collected by hypochlorite treatment and allowed to hatch overnight at 20°C in 1x M9 buffer. L1 larvae were then plated on 6 cm NGM plates freshly seeded with 10x concentrated *E. coli* OP50 bacteria, and allowed to develop into 1-day old adult worms at 20°C. By picking, 200 adult worms were collected into sterile water and flash frozen in liquid nitrogen for each genotype/sample. Each frozen sample then had 2x Laemmli Buffer added in equal volume and boiled. Samples were centrifuged at 4°C and the collected supernatants were used for electrophoresis. Equivalent volumes samples were run on pre-cast 4-20% polyacrylamide gels (Bio-Rad, Cat #4561093) and then transferred to PVDF membranes (Trans-Blot^®^ Turbo™ Midi PVDF Transfer Packs, Cat #1704157) using the Trans-Turbo Blot system (Bio-Rad). Membranes were blocked in 5% milk at room temperature, then exposed to the primary p-CREB S133 antibody (Abcam, Cat #Ab32096, 1:750) or beta-actin antibody (EMD, Cat #Mab1501r, 1:3,000 dilution) diluted in 5% milk at 4°C overnight. After overnight incubation, membranes were exposed to HRP-conjugated secondary antibody, anti-rabbit (Cell Signaling Technologies, Cat #7074s, 1:5,000 dilution) or anti-mouse (Cell Signaling Technologies, Cat # 7076s, 1:5,000 dilution), diluted in 5% milk. Membranes were then exposed to ECL (Clarity™ Western ECL Substrate, Cat #1705060), and visualized and captured with the ChemiDoc V3 Touch System (Bio-Rad). Relative quantities of p-CREB and actin bands were determined by ImageJ version 1.53 (NIH) as described by (Gallo-Oller et al., 2018).

**Lifespan analysis**

All strains were maintained at 20°C for at least two generations before the lifespan assay. Adult worms age-synchronized by hypochlorite treatment were allowed to lay eggs on NGM plates seeded with 10x concentrated *E. coli* OP50 bacteria over ~3 hours, and then removed. The resulting progeny synchronized by the timed egg-laying were allowed to develop into L4 larvae at 20°C. At the L4 stage, 100-200 worms per genotype were transferred to new 6 cm NGM plates with freshly seeded 10x concentrated *E. coli* OP50 bacteria. Wild-type controls (N2) were assayed in parallel to mutants in the absence of FUdR and at 20°C. Adult worms were transferred every 2 days during active reproduction, and each plate contained 10-15 worms. Worms that experienced ventral rupture, bagging, or walling were censored from the life-span analysis. A worm was considered dead when it did not respond to touch of the platinum wire pick, and was subsequently removed from the plate.

**Statistical analysis**

Statistical comparisons and graphical representations for lifespan curves were performed with the Online Application for Survival Analysis (OASIS2,<https://sbi.postech.ac.kr/oasis2/> (Han et al., 2016). Lifespan curves were analyzed by a log-rank Mantel-Cox test. Other data were analyzed using Graphpad Prism 9 software. Statistical comparisons made include the Mann-Whitney *t*-test or the one-way ANOVA followed by a posthoc multiple-comparisons test. *P* values are reported in the figure legends.

Reference List

Brenner, S. (1974). The genetics of Caenorhabditis elegans. Genetics *77*, 71-94.

Cortes-Lopez, M., Gruner, M.R., Cooper, D.A., Gruner, H.N., Voda, A.I., van der Linden, A.M., and Miura, P. (2018). Global accumulation of circRNAs during aging in Caenorhabditis elegans. BMC Genomics *19*, 8.

Gallo-Oller, G., Ordonez, R., and Dotor, J. (2018). A new background subtraction method for Western blot densitometry band quantification through image analysis software. J Immunol Methods *457*, 1-5.

Han, S.K., Lee, D., Lee, H., Kim, D., Son, H.G., Yang, J.S., Lee, S.V., and Kim, S. (2016). OASIS 2: online application for survival analysis 2 with features for the analysis of maximal lifespan and healthspan in aging research. Oncotarget *7*, 56147-56152.

Huang da, W., Sherman, B.T., and Lempicki, R.A. (2009). Systematic and integrative analysis of large gene lists using DAVID bioinformatics resources. Nat Protoc *4*, 44-57.
